# Neuropeptide Y regulates cholesterol uptake and efflux in macrophages and promotes foam cell formation

**DOI:** 10.1111/jcmm.17561

**Published:** 2022-09-29

**Authors:** Yu Cai, Zhengchao Wang, Lun Li, Li He, Xinying Wu, Mingjing Zhang, Pengfei Zhu

**Affiliations:** ^1^ Department of Rehabilitation Wuhan Fourth Hospital Wuhan China; ^2^ Department of Orthopedics, Tongji Medical College, Tongji Hospital Huazhong University of Science and Technology Wuhan China; ^3^ Department of Cardiology Wuhan Fourth Hospital Wuhan China

**Keywords:** foam cell, macrophage, NPY, THP‐1

## Abstract

The dysregulation of lipid metabolic pathways (cholesterol uptake and efflux) in macrophages results in the formation of lipid‐dense macrophages, named foam cells, that participate in plaque formation. NPY binding to NPY receptors in macrophages can modulate cell functions and affect the process of atherosclerotic plaques. The present study aimed to determine whether NPY affects the formation of macrophage‐derived foam cells and its underlying mechanisms in macrophages. THP‐1‐derived macrophages were incubated with oxidized low‐density lipoprotein (ox‐LDL) and treated with different concentrations of NPY. We analysed the relative levels of proteins related to cholesterol uptake and efflux. We found that NPY effectively increased cholesterol uptake and intracellular cholesterol content via the Y1 and Y5 receptors, and this effect was blocked by Y1 and Y5 antagonists. Mechanistically, NPY enhanced the expression of SRA and CD36 via the PKC/PPARγ pathways, promoting macrophage cholesterol uptake. Moreover, NPY significantly decreased cholesterol efflux to the extracellular cholesterol acceptors ApoA1 and HDL in macrophages. NPY mediated decreases in ABCA1, ABCG1 and SR‐BI expression through the inhibition of the JAK/STAT3 pathways. Our results suggest that NPY binding to the Y1 and Y5 receptors enhances foam cell formation by regulating cholesterol uptake and efflux in macrophages.

## 1 INTRODUCTION

The formation and accumulation of foam cells in the artery wall contribute to the occurrence of atherosclerotic plaques, in which foam cells formed by macrophage phagocytosis of lipids are a crucial part.^1^ Under abnormal circumstances, the three processes of lipid metabolism by macrophages, cholesterol uptake, esterification and efflux, are disturbed and in turn result in macrophage‐derived foam cells. Several extracellular substances participate in triggering foam cell formation, especially excessive oxidized low‐density lipoprotein (ox‐LDL), which exceeds the digestive capacity of macrophages and leads to the formation of macrophage‐derived foam cells.^2^

Macrophage scavenger receptor class A (SRA) and CD36 play the most important roles in regulating ox‐LDL uptake and are responsible for foam cell formation.^3^, ^4^ SRA and CD36 accounted for 75%–90% of ox‐LDL internalization by macrophages.^5^ Studies have proven that SRA and CD36 inhibition can significantly decrease foam cell formation and alleviate atherosclerotic plaques.^5^, ^6^ The cholesterol metabolism in macrophages is related to the balance of cholesterol ester and cholesterol efflux following cellular uptake. Free cholesterol (FC) was stored as cholesterol esters by the enzyme acylcholesterol transferase 1 (ACAT‐1) after the accumulation of FC in the cells. ACAT1 is the rate‐limiting protein for cholesterol ester processes,^7^ and increased ACAT1 expression accelerates cholesterol accumulation in macrophages.^8^ However, studies attempting to inhibit foam cell formation by targeting ACAT‐1 have not been successful. Both ACAT‐1 knock down or pharmacological inhibition of ACAT‐1 did not show atherosclerosis lesion and foam cell formation reduction.^9^, ^10^ Cholesterol reverse transport is an essential stage in macrophage‐mediated plasma lipoprotein metabolism. For cholesterol efflux processes, ATP‐binding cassette transporter 1 (ABCA1) and ATP‐binding cassette subfamily G member‐1 (ABCG1) are the key proteins in cholesterol efflux and are mainly responsible for facilitating cholesterol removal from macrophages and preventing excessive intracellular cholesterol accumulation.^11^ Studies revealed that reduced ABCA1 or ABCG1 mediated cholesterol efflux promoted plaque formation and accelerated atherosclerosis.^12^, ^13^, ^14^ Scavenger receptor class B type I (SR‐BI) is responsible for transporting cholesterol to HDL. Inhibition of plaque formation by SR‐BI overproduction and plaque growth enhancement by depletion of SR‐BI in macrophages indicated the anti‐atherogenic role of this scavenger receptor.^15^, ^16^ Therefore, ABCA1, ABCG1 and SR‐BI play a critical role in foam cell formation prevention and protect against atherosclerotic lesions.^11^, ^12^, ^13^, ^14^, ^15^, ^16^

Neuropeptide Y (NPY), a neurotransmitter, is abundantly distributed in the peripheral nervous system and was shown to be increased in the artery wall and be related to atherosclerosis.^17^, ^18^, ^19^ Studies also found that the increase in NPY is accompanied by the activation of the G protein‐coupled Y‐family of receptors 1 and 5 (Y1R and Y5R) in atherosclerosis.^17^, ^18^, ^20^ Similarly, evidence for NPY‐mediated effects on macrophages has arisen from numerous studies showing the contributions of NPY in modulating macrophage functions via NPY‐Y1 and NYP‐Y2/Y5 receptors.^21^, ^22^, ^23^ Since foam formation in the artery wall is inseparable from the role of macrophages and NPY not only enhances the process of atherosclerosis but also influences the function of macrophages, the relationships of NPY, macrophages and atherosclerotic plaques attracted our attention. In a rat model of balloon injury‐induced artery plaque, the expression of Y1R, Y2R and Y5R in macrophages increased after NPY administration to injured regions of the carotid artery.^24^ This finding suggested that NPY may activate Y1R, Y2R or Y5R in macrophages and modulate foam cell formation, thus participating in the process of atherosclerotic plaques.

As mentioned above, CD36 and SRA promote cholesterol uptake, and ABCA1, ABCG1 and SR‐BI facilitate cholesterol removal from macrophages. Whether NPY induction participates in the regulation of these proteins remains unclear. This study presents insight into the effect of NPY and NPY‐1/Y2/Y5 on foam formation and investigates the regulation of cholesterol uptake and cholesterol efflux by NPY.

## 2 MATERIALS AND METHODS

### 2.1 Reagents and antibodies

DMEM was purchased from Gibco (Life Technologies). Human copper‐oxidized low‐density lipoprotein (ox‐LDL) and DiI‐Ox‐LDL were obtained from Biomedical Technologies. NPY (ab120208), Y1, Y2 and Y5 antagonists were obtained from Sigma. HDL (L8039) and apolipoprotein A‐I (ApoA‐I, SRP4693) were obtained from Sigma.

Anti‐PKC (SAB4502354), Anti‐p‐PKC (SAB4301254), anti‐JAK2 (SAB4501601) and anti‐p‐JAK‐2 (SAB4300124) antibodies were purchased from Sigma. AntiABCA1 (NB400‐105) and antiABCG1 (NB400‐132) antibodies were purchased from Novus Biologicals, Inc. PPARγ, anti‐CD36 (sc‐9154), anti‐SRA and anti‐β‐actin were obtained from Santa Cruz Biotechnology, Inc.

### 2.2 Macrophages deriving

THP‐1 cells were purchased from Procell Life Science & Technology Co., Ltd (CL‐0233). The cells were incubated and passaged according to the instruction. As for the macrophage deriving, the cells were cultured in RPMI 1640 containing 10% FBS and 100 U/ml penicillin/streptomycin (at a concentration of 1 × 10^6^ cells/ml) and stimulated with 160 nmol/L PMA for 24 h in 6‐well plates. After starvation with serum for 12 h, the cells were treated with vehicle control (PBS), ox‐LDL (80 μg/ml) or NPY (0.5, 1 and 2 μM). After 24 h of incubation, the adherent cells were collected for Oil Red O staining or cholesterol measurement.

### 2.3 Cell viability assay

THP‐1‐derived macrophages were seeded in black 96‐well plates and incubated until they reached 80% confluence. Confluent cells were treated with or without NPY (1.0 μM) for the indicated time. Then, cell viability was measured by a Cell Counting Kit‐8 (CCK‐8; Dongin Biotech).

### 2.4 Oil Red O staining

Upon fixation with paraformaldehyde (4%), the cells were stained with 0.3% Oil Red O for 15 min. Haematoxylin was used as a counterstain and evaluated via microscopy. The stained cells were eluted with isopropanol, and the supernatant was collected; the OD of the extracts was measured at 540 nm.

### 2.5 Measurement of cholesterol contents

The cultured cells were lysed by sonicating the cells in hexane/isopropanol. After centrifugation at 10,000 g, the supernatant was collected to determine intracellular cholesterol. Total cholesterol and free cholesterol were detected using an Amplex® Red Cholesterol Assay kit (Life Technologies). The cholesterol ester content was determined by subtracting free cholesterol from total cholesterol. The cholesterol content was expressed as follows:

$$\begin{array}{c} \text{Cholesterol concentration}\;\left(\mathrm{\mu g}/\mathrm{\mu l}\right)=\\ \;\text{intracellular cholesterol}\;\left(\mathrm{\mu g}\right)/\text{sample volume} \end{array}$$

### 2.6 DiI‐ox‐LDL uptake

Macrophages were seeded on confocal dishes at a density of 5 × 10^5^ cells/ml and incubated with vehicle control (PBS containing 0.1% DMSO) or NPY (0.5, 1, and 2 μM) for 12 h. Then, the cells were incubated with DiI‐ox‐LDL for 4 h. After being fixed in 4% formalin for 10 min, the cells were detected and analysed by confocal microscopy.

### 2.7 Cholesterol efflux assay

Macrophage‐specific cholesterol efflux capacity was measured using a commercial available Cholesterol Efflux Assay Kit (Abcam, ab196985). Briefly, macrophages (1 × 10^5^) were labelled with fluorescently labelled cholesterol and equilibrated for 24 h in a humidified incubator at 37°C and 5% CO_2_. After two washes, the collected cells were recultured with NPY (0.5, 1 and 2 μM) for 24 h. Cells were incubated in DMEM containing HDL or ApoA1 for 6 h again. The cholesterol contents in the medium and cell lysates were measured. The cholesterol efflux to HDL or ApoA1 was expressed as a percentage of fluorescence in the medium and cell lysis relative to the total amount of fluorescence.

### 2.8 Real‐time polymerase chain reaction

Total RNA was extracted using a Total RNA kit (TaKaRa) according to the manufacturer's instructions. The purity and concentrations of RNA were detected using a spectrophotometer. Reverse transcription and cDNA synthesis were performed using an RNA PCR kit (TaKaRa). Real‐time polymerase chain reaction was performed using a Step One SYBR Green Mix Kit (TaKaRa) and ABI Prism Sequence Detection System (Applied Biosystems) according to the manufacturer's instructions. The housekeeping gene GAPDH was used as an internal control. The conditions of the amplification reaction were 95°C for 30 s, 95°C for 5 s and 60°C for 30 s, and PCR was performed for 40 cycles. The relative PCR primers are shown in Table 1. The target gene mRNA expression was calculated using the 2^−ΔΔ*C*t^ method.

**TABLE 1:** Primer sequences of RT‐PCR

Genes	Sequence (5′‐3′)
Y1	F: 5′‐CTTGCTGGTCGCAGTCATGT‐3′
	R: 5′‐ GAAGAGTCGTGTAAGACAGCC‐3′
Y2	F: 5′‐AACTTGCTGTGGACATCGACAG‐3′
	R: 5′‐GCTAGGACACCTCCGAGTGA‐3′
Y5	F: 5′‐GGGACAATCCACAGCTTATGC‐3
	R: 5′‐AAATCGTCTACGCTGCCTCTG‐3′
SRA	F: 5′‐CAAACGCACTCCCCTTACTA‐3′
	R: 5′‐TGTTCCAGAGGTCTCAGCAC‐3′
CD36	F: 5′‐GAGGATGTCAATGGCTTTCA‐3′
	R: 5′‐TCATTTTTCTAATAACAAAA‐3′
ABCA1	F: 5′‐GATGGCAATCATGGTCAATGG‐3′
	R: 5′‐AGCTGGTATTGTAGCATGTYCCG‐3′
ABCG1	F: 5′‐GGG GTCGCTCCATCATTTG‐3′
	R: 5′‐TTCCCCGGTACACACATTGTC‐3′

### 2.9 Protein extraction and Western blot

After intervention, the cells were collected and washed twice with cold PBS. Nuclear and cytoplasmic proteins were extracted using NE‐PER Nuclear and Cytoplasmic Extraction Reagents (Thermo Scientific) according to the manufacturer's instructions. The Lowry assay was used to estimate the protein concentration. Equal amounts of sample proteins were resolved on SDS‐polyacrylamide (SDS‐PAGE) gels and then electrophoretically transferred onto a nylon‐enhanced nitrocellulose membrane. After blocking with 5% nonfat milk protein for 2 h, the membranes were incubated with primary antibodies at 4°C overnight. Next, the membranes were incubated with HRP‐conjugated IgG secondary antibody at room temperature for 2 h. The immunoreactive bands were obtained, and the images were captured and semiquantitatively analysed by Quantity One software (Bio‐Rad).

### 2.10 Immunofluorescence

The cell climbing sheets (adherent macrophage cells grow on the cover glass) were washed twice with phosphate buffered saline (PBS) and fixed with 4% polyformaldehyde for 30 min. Then, the cells were washed twice with PBS buffer containing 0.5% Triton X‐100 for 20 min and blocked with 5% bovine serum albumin (BSA) in PBS buffer for 10 min. The climbing sheets were incubated with primary antibodies (SRA [1:1000, rabbit, CST] or CD36 [1:1000, rabbit, Abcam]) for 30 min at 37°C. After three washes with PBS, the climbing sheets were incubated with DyLight 594‐conjugated goat anti‐rabbit IgG antibodies (1:500) at 37°C for 30 min. After two washes with PBS and nuclear staining with DAPI, fluorescence staining was visualized using a fluorescence microscope (Olympus, IX71), and fluorescence intensity was analysed with IPP software as the means of the ratio of total intensity and area of visual field. All experiments were performed in biological triplicates, and the data are representative of at least three independent experiments.

### 2.11 Statistical analysis

Statistical analyses were performed using SPSS 21.0 software. The normality of distribution was tested by a QQ plot. When data were normally distributed and group variances were equal, one‐way anova was used for multiple comparisons. When group data were not normally distributed or if group variances were unequal, the Kruskal–Wallis test was used. A post hoc test of the *p* value was performed by Bonferroni correction. A value of *p* < 0.05 was considered statistically significant.

## 3 RESULTS

### 3.1 NPY increased ox‐LDL‐induced cholesterol contents and promoted foam cell formation

**FIGURE 1:**
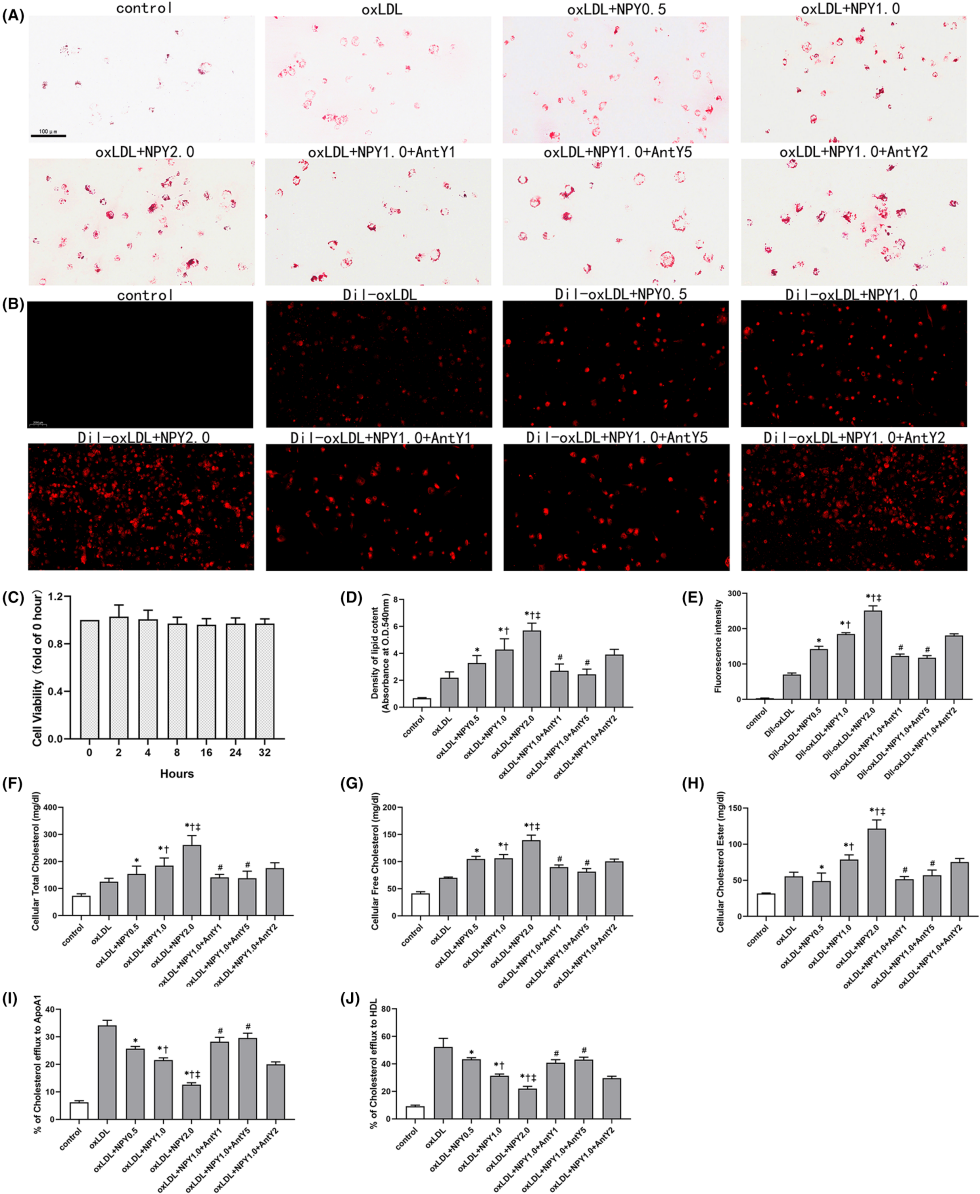
NPY significantly enhance the uptake of oxLDL and Dil‐oxLDL and foam formation in macrophage under different concentration. (A) Macrophages were treated with oxLDL (50 μg/ml) in the presence or absence of NPY (0.5, 1.0 and 2.0 μM) and NPY receptor antagonists. After treatment, the cells were stained with Oil Red O. (B) Macrophages were incubated with Dil‐oxLDL (10 μg/ml) in the presence or absence of NPY (0.5, 1.0 and 2.0 μM) and NPY receptor antagonists for 4 h. (C) Macrophages were treated with NPY (2.0 μM) for 2, 4, 8, 16, 24 and 32 h, and cell viability was measured by CCK‐8 assay. (D) The Oil Red O stained was extracted, and the lipid contents in macrophages were expressed by the absorbance at 540 nm. (E) Dil‐oxLDL uptake was evaluated by immunofluorescence analysis. Macrophages were treated as described above, and cellular total cholesterol (F), free cholesterol (G) and cholesteryl ester (H) were measured as described in the Methods section. (I) and (J) ApoA1 and HDL mediated cholesterol was measured under NPY with or without NPY receptor antagonists. Data were expressed as mean ± SEM. *p < 0.05 compared to the oxLDL; †*p* < 0.05 compared to oxLDL+NPY0.5; ‡*p* < 0.05 compared to oxLDL+NPY1.0; #*p* < 0.05 compared to oxLDL+NPY1.0

First, we evaluated the effect of NPY on macrophage viability and found NPY at 2.0 μM was not toxic to macrophages for up to 32 h (Figure 1C). To explore the effects of NPY on ox‐LDL uptake and foam cell formation, we incubated macrophages with NPY (0.5, 1.0, 2.0 μM) for 12 h. After two washes with PBS, the cells were then incubated with DiI‐ox‐LDL (10 μg/ml) for 4 h or ox‐LDL (50 μg/ml) for 12 h. Macrophages were loaded with ox‐LDL + NPY, and Oil Red O staining was performed. The results showed that NPY significantly accelerated foam cell formation (Figure 1A,D). Macrophages were loaded with DiI‐ox‐LDL, and immunofluorescence analysis was performed. The results showed that NPY treatment markedly facilitated DiI‐ox‐LDL uptake in macrophages (Figure 1B,E). We further examined the effect of NPY on the cholesterol contents of the ox‐LDL‐treated macrophages by analysing the intracellular total cholesterol content, free cholesterol content and cholesteryl ester (Figure 1F–H). The results showed that NPY dose‐dependently increased cellular cholesterol levels (Figure 1F–H). These data demonstrate that NPY effectively enhances macrophage foam cell formation by increasing cholesterol uptake.

### 3.2 NPY binding to Y1R/Y5R mediates foam cell formation

**FIGURE 2:**
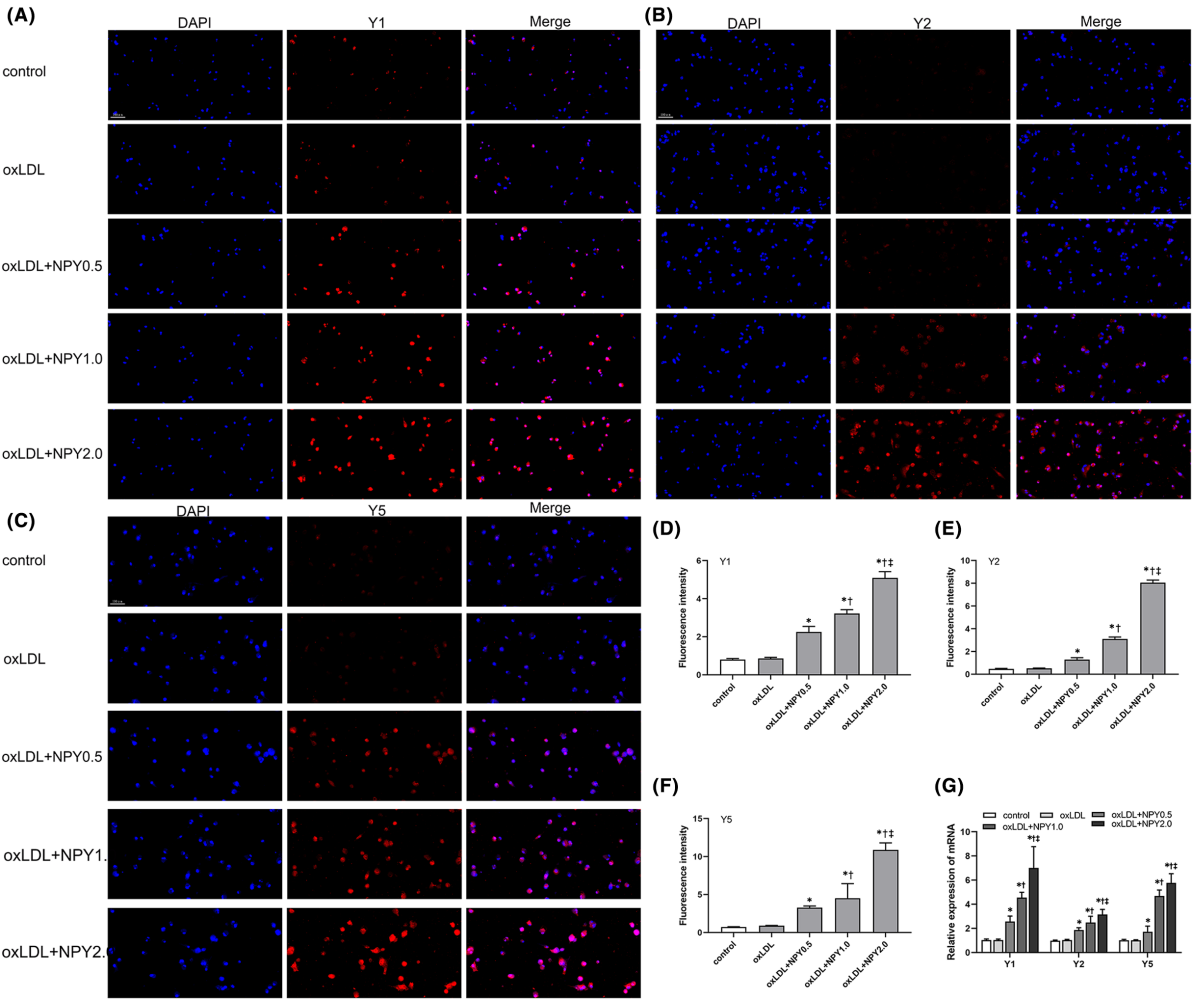
Effects of NPY on the expression of NPY receptors (Y1, Y2 and Y5). (A–C) Macrophages were treated with oxLDL (50 μg/ml) in the presence or absence of NPY (1.0 μM) and NPY receptors antagonist. Y1, Y2 and Y5 were detected by immunofluorescence staining. (D–F) The results of fluorescence intensity showed that NPY promoted the expression of Y1, Y2 and Y5 in a dose‐dependent manner. (G) The mRNA levels of Y1, Y2 and Y5 of oxLDL‐treated macrophages in different concentrations of NPY (0.5, 1.0 and 2.0 μM). Data were expressed as mean ± SEM. *p < 0.05 compared to oxLDL; †*p* < 0.05 compared to oxLDL+NPY0.5; ‡*p* < 0.05 compared to oxLDL+NPY1.0

The Y1, Y2 and Y5 receptors were all expressed in macrophages and increased in the plaque area.^24^ We detected the expression of the Y1, Y2 and Y5 receptors in the NPY‐treated ox‐LDL‐treated macrophages by immunofluorescence staining (Figure 2A–C) and RT‐PCR. Analysis of mRNA showed that the levels of the Y1, Y2 and Y5 receptors were all upregulated by NPY in a dose‐dependent manner (Figure 2G). The results were consistent with immunofluorescence analysis (Figure 2D–F). To further determine whether the upregulated expression of Y1, Y2 and Y5 receptors was associated with cholesterol uptake, we incubated ox‐LDL‐ or DiI‐ox‐LDL‐induced macrophages with NPY and NPY receptor antagonists. Notably, Y1 and Y5 antagonists inhibited the uptake of cholesterol and reversed the inhibition of NPY on ApoA1‐ and HD‐specific cholesterol efflux (Figure 1I,J). However, the Y2 antagonist did not have similar effects (Figure 1I,J). This result indicated that NPY binding to Y1/Y5 receptors participated in cholesterol transport and may influence foam cell formation.

### 3.3 NPY regulates the expression of SRA and CD36

**FIGURE 3:**
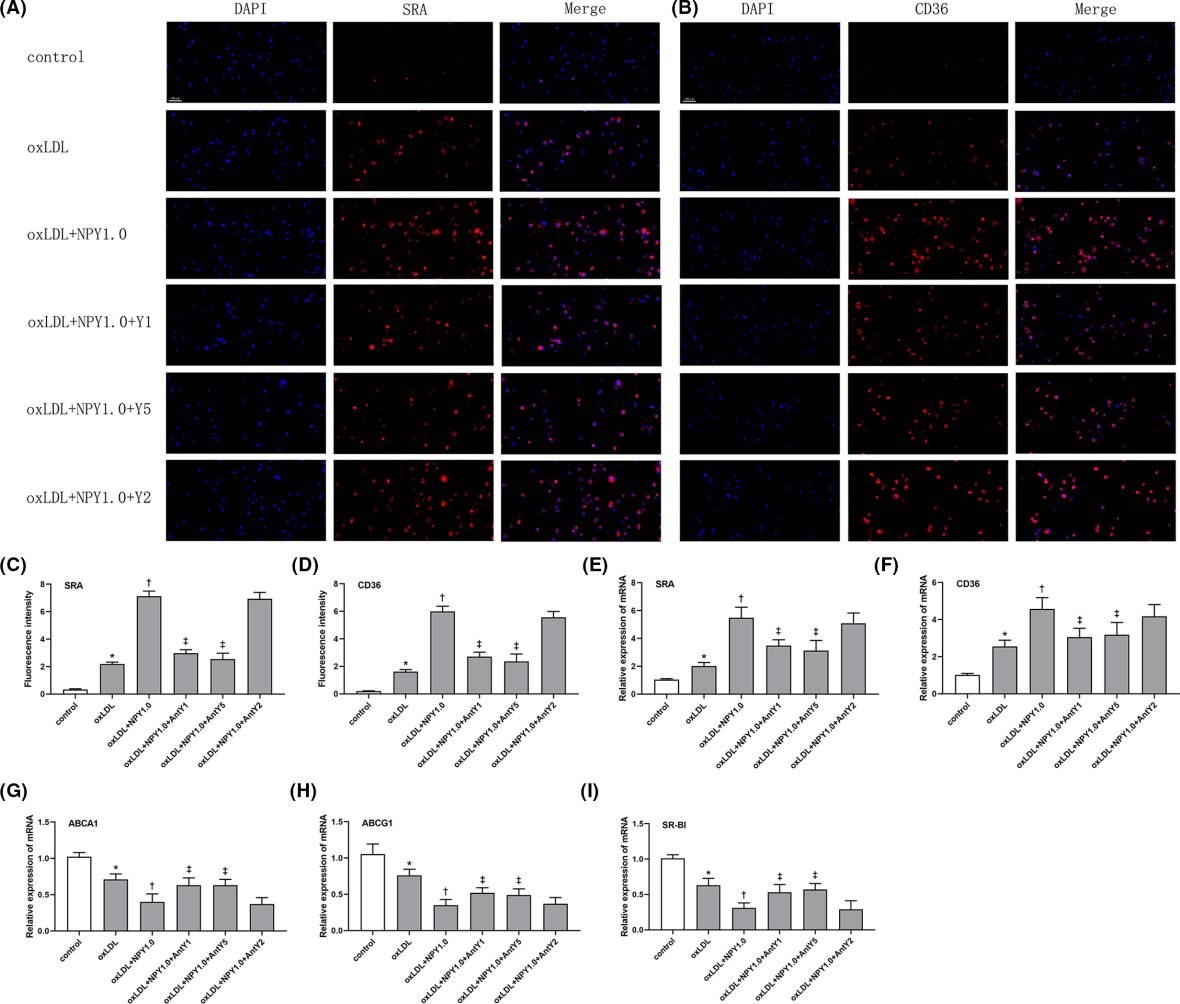
Effects of NPY (1.0 μM) on the expression of SRA and CD36 in oxLDL‐treated macrophages. (A, B) Macrophages were treated with oxLDL (50 μg/ml) in the presence or absence of NPY (1.0 μM) and NPY receptors antagonist. SRA and CD36 were detected by immunofluorescence staining. (C, D) The levels of SRA and CD36 were presented as the fluorescence intensity. (E–H) The mRNA levels of SRA, CD36, ACBA1 and ACBG1 in oxLDL‐treated macrophages in different concentrations of NPY (0.5, 1.0 and 2.0 μM). Data were expressed as mean ± SEM. *p < 0.05 compared to the control; †*p* < 0.05 compared to oxLDL; ‡*p* < 0.05 compared to oxLDL+NPY1.0

**FIGURE 4:**
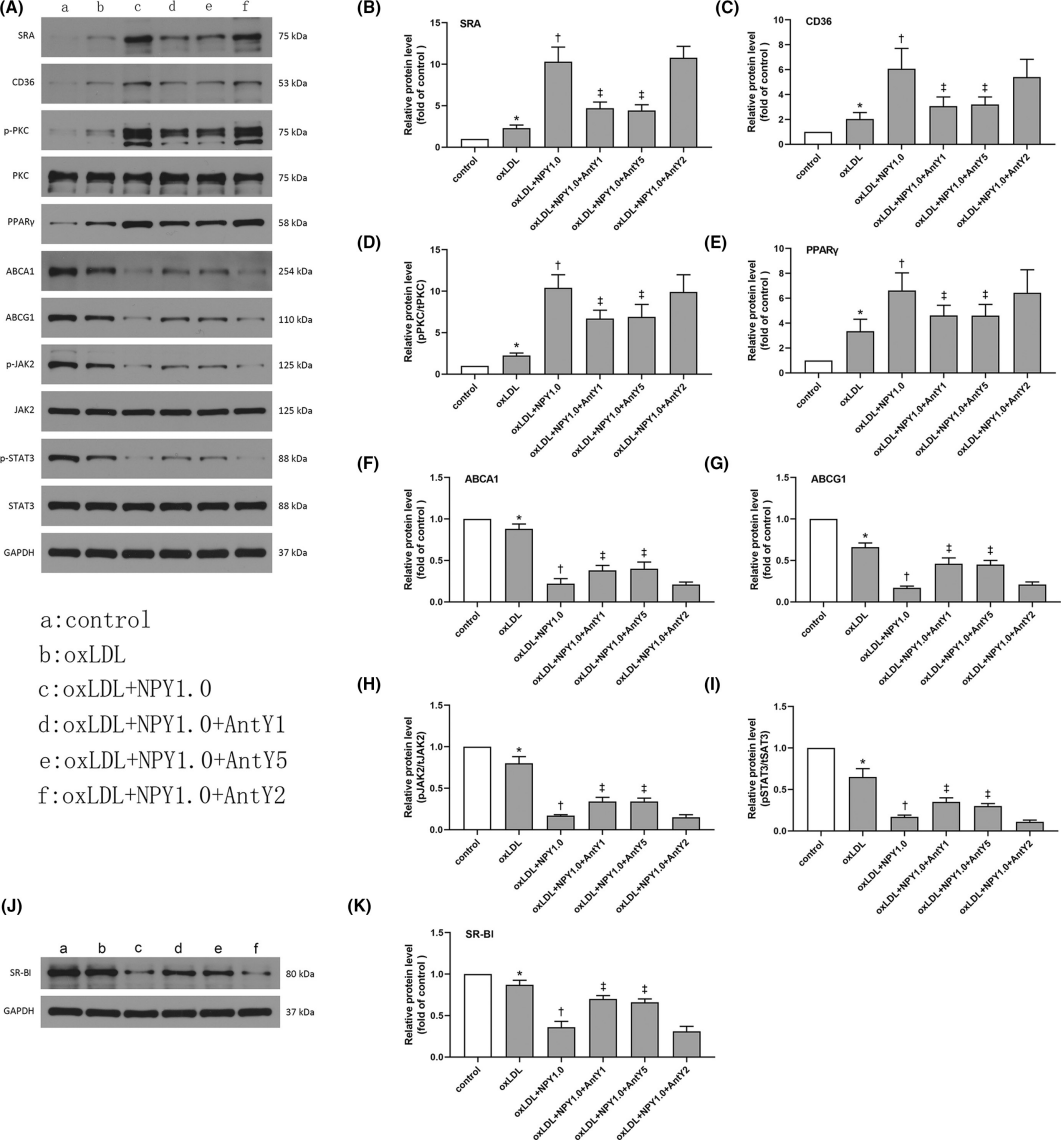
Effects of NPY (1.0 μM) on the protein levels of scavenger receptors and cholesterol transports in oxLDL‐treated macrophages. (A) The oxLDL‐treated macrophages were incubated with NPY (1.0 μM) or NPY receptors antagonist. Cell lysates were subjected to Western blot to assess the protein levels of SRA, CD36, ACBA1 and ACBG1. The protein levels of phospho‐PKC, phospho‐JAK2 and phospho‐STAT3 were also measured by Western blot. Nuclear proteins were isolated, and PPARγ protein levels were determined by Western blot. Data were expressed as mean ± SEM. (B–E) NPY enhanced the levels of SRA and CD36 by up‐regulating PKC/PPARγ signal pathway. (F–I) NPY inhibited the levels of ACBA1 and ACBG1 by downregulating JAK2/STAT3 signal pathway. Data were expressed as mean ± SEM. (J, K) The oxLDL‐treated macrophages were incubated with NPY (1.0 μM) or NPY receptors antagonist. Cell lysates were subjected to Western blot to assess the protein levels of SR‐BI were also measured by Western blot. NPY inhibited the levels of SR‐BI by downregulating JAK2/STAT3 signal pathway. Data were expressed as mean ± SEM. *p < 0.05 compared to control; †*p* < 0.05 compared to oxLDL; ‡*p* < 0.05 compared to oxLDL+NPY1.0

Because cholesterol uptake by macrophages is mediated by scavenger receptors expressed on macrophages, we evaluated the effect of NPY on the expression of CD36 and SRA in NPY‐induced foam cell formation. The immunofluorescence results demonstrated that NPY could increase the expression of SRA and CD36 (Figure 3A–D). As shown in Figures 3E,F and 4A–C, treatment with NPY caused an obvious increase in the mRNA and protein levels of SRA and CD36. These effects also could be reversed by Y1 and Y5 antagonists which illustrated that NPY enhanced the expression of SRA and CD36 by Y1 and Y5 receptors (Figure 3A–F). These data demonstrate that NPY binding to Y1 and Y5 receptors enhances macrophage foam cell formation by increasing cholesterol uptake via upregulation of SRA and CD36 expression.

### 3.4 NPY decreases cholesterol efflux by downregulating ABCA1, ABCG1 and SR‐BI expression

Research findings proved that ABCA1, ABCG1 and SR‐BI are key transporters that facilitate cholesterol egress from macrophages, reducing atherosclerotic development.^13^, ^14^, ^15^ We detected the key transporters responsible for cholesterol efflux, ABCA1, ABCG1 and SR‐BI, under NPY conditions. The results showed that NPY treatment decreased the expression of ABCA1, ABCG1 and SR‐BI at the mRNA and protein levels (Figure 3G–I).

Cholesterol efflux is achieved through extracellular cholesterol acceptors such as ApoA1 and HDL. We investigated whether the decreases in ABCA1, ABCG1 and SR‐BI mediated by NPY impacted the cholesterol efflux efficiency in macrophages. As shown in Figures 1I,J and 3G–I, NPY significantly inhibited cholesterol efflux to the extracellular cholesterol acceptors ApoA1 and HDL in macrophages by downregulating the expression of ABCA1, ABCG1 and SR‐BI suggesting that NPY decreases cholesterol efflux, resulting in an increase in foam cell formation.

### 3.5 NPY mediated SRA and CD36 expression via the pPKC/PPARγ signalling pathway

In this study, we found that pPKC expression was obviously increased at protein levels in the ox‐LDL‐induced macrophages with or without NPY conditions (Figure 4A,D). Furthermore, the activation of the pPKC /PPARγ signalling pathways was proven to lead to the induction of CD36 and SRA, which are involved in ox‐LDL uptake.^25^ Consistent with a previous study, PPARγ expression was increased in the ox‐LDL‐treated macrophages, and this effect was enhanced by NPY treatment (Figure 4A,E). The effects of NPY on the regulation of pPKC/PPARγ were inhibited by both Y1 and Y5 antagonists. Our results showed that NPY‐induced pPKC expression was decreased by 35.6% by the Y1 receptor antagonist and by 34.1% by the Y5 antagonist (Figure 4A,D), and PPARγ expression was decreased by 30.6% by the Y1 receptor antagonist and by 31.3% by the Y5 antagonist.

### 3.6 NPY inhibited ABCA1, ABCG1 and SR‐BI expression via the JAK2/STAT3 signalling pathway

It has been proven that the JAK2/STAT3 pathway upregulates the levels of ABCA1, ABCG1 and SR‐BI and mediates cholesterol efflux in macrophages.^26^ In this study, we found a reduction in ABCA1, ABCG1 and SR‐BI and downregulation of JAK2/STAT3 phosphorylation in the ox‐LDL‐treated macrophages (Figure 4A,F–K), which was consistent with a previous study.^26^ Next, we investigated whether the JAK2/STAT3 pathway is also involved in NPY‐mediated ABCA1, ABCG1 and SR‐BI expression. NPY treatment strongly downregulated ABCA1, ABCG1 and SR‐BI expression and therefore inhibited cholesterol efflux (Figure 4A,F–K). The JAK2/STAT3 pathway was also inhibited by NPY treatment. The inhibitory effect of NPY on ABCA1, ABCG1 and SR‐BI expression and the JAK2/STAT3 pathway could be reversed by Y1 and Y5 antagonists to some degree (Figure 4A,F–K). These data demonstrated that NPY decreased the expression of ABCA1, ABCG1 and SR‐BI by inhibiting the JAK2/STAT3 pathway and therefore inhibited cholesterol efflux.

## 4 DISCUSSION

During the progression of atherosclerotic plaques, deposition of excessive lipids into the damaged subendothelium in arteries followed by macrophage uptake and foam cell formation results in the characteristic fatty streak in the early stages of atherosclerosis. Formation of foam cells within the lesion area via ox‐LDL uptake by macrophages initiates various inflammatory responses and cell death and is influenced by other factors. In this study, we found that NPY significantly enhanced the uptake of ox‐LDL in macrophages by binding to Y1R/Y5R. The promoting effect of foam cell formation is mediated at least in part via induction of SRA and CD36 and inhibition of ABCA1, ABCG1 and SR‐BI.

Atherosclerotic plaque formation and disease progression are associated with the sympathetic nervous system. Sympathetic activity affects atherosclerosis mainly via its transmitters for noradrenaline (NE), ATP and neuropeptide Y (NPY). A recent study found that NE appears to play a role in foam cell formation by preventing cholesterol efflux from macrophages by promoting ABCA1 degradation.^27^ NPY, as a cotransmitter, is mainly found in postganglionic sympathetic neurons and is released simultaneously with norepinephrine (NE) in response to sympathetic stimulation.^28^ NPY has a much longer half‐life and duration of action^29^ and is involved in atherosclerosis by acting with endothelial cells, macrophages, vascular smooth muscle cells and platelets.^19^ Li et al.^24^ generated a rat model of balloon injury arteriosclerosis with the administration of NPY to local injured regions and found numerous macrophages with the Y1, Y2 and Y5 receptors of NPY on atheromatous plaque lesions. Similarly, Li et al.^17^ investigated the NPY system in patients with peripheral artery diseases (PAD) and showed that upregulation of the expression of NPY or its receptors is associated with human atherosclerotic lesion burden and vulnerability.

Studies in vitro have proven that NPY modulates various functions of macrophages, such as adherence capacity, chemotaxis and phagocytosis.^22^, ^30^, ^31^, ^32^ NPY increased the oxidative burst of phorbol myristate acetate (PMA)‐stimulated or zymosan‐stimulated macrophages by interacting with Y1, Y2 and Y5.^31^

The above evidence suggests that NPY has an important role in plaque formation, such as modulating macrophage foam cell formation. Therefore, we treated the ox‐LDL‐treated macrophages with different concentrations of NPY. The results showed that NPY promoted the formation of foam cells in a concentration‐dependent manner in vitro. Because the three receptors Y1, Y2 and Y5 were detected in plaque areas, we next investigated the expression of these three receptors in NPY‐induced foam cell formation. Our study found that the Y2 antagonist alone did not affect foam cell formation, as shown in Figure 1. In contrast to the above result, both the Y1 and Y5 antagonists inhibited NPY‐induced foam cell formation by 30%. Even more remarkably, the effect was entirely inhibited when treated with the combination of Y1 and Y5 antagonists. It seems that the Y1 and Y5 receptors play a major role in NPY‐induced foam cell formation.

Either increased cholesterol uptake or reduced cholesterol efflux enhanced the formation of macrophage‐derived foam cells. In the cholesterol uptake process, CD36 and SRA are recognized as vital regulatory factors. Our in vitro foam cell assay found that NPY enhanced foam cell formation by increasing DiI‐ox‐LDL uptake in THP‐1‐derived macrophages. To further investigate whether SRA and CD36 were required for this process, we detected the expression of SRA and CD36 in the ox‐LDL‐treated macrophages treated with different concentrations of NPY. A dose‐dependent increase in the mRNA and protein levels of SRA and CD36 was observed with increased concentrations of NPY. The cholesterol efflux of macrophages maintains cell homeostasis mainly by membrane transporters, such as ABCA1 and ABCG1.^7^, ^11^, ^12^ Foam cell formation has close relationships with cholesterol efflux blockade and downregulation of transporter expression. Interestingly, the expression of ABCA1 and ABCG1 was evidently inhibited by NPY in this study. The results revealed that NPY influenced the process of cholesterol uptake and cholesterol efflux and mediated foam cell formation.

Moreover, the stimulatory functions of NPY on macrophages are mediated by the PKC signalling pathway.^31^, ^33^ Zhou et al.^34^ also found that NPY binding to Y1 promoted the synthesis and release of high mobility group protein B1 through the PKC/ERK pathway. In an in vitro study of PMA‐stimulated macrophages, NPY was proven to enhance the oxidative burst through a PKC‐independent mechanism.^31^ In this study, we showed that NPY upregulated the expression of PKC by Y1 and Y5 in the ox‐LDL‐stimulated macrophages. The upregulation of PKC expression was followed by increased levels of SRA and CD36, which was consistent with a previous study.^35^ This finding demonstrated that NPY binding to Y1 and Y5 activated PKC and regulated the expression of SRA and CD36.

A previous study proved that SRA and CD36 expression in macrophages interferes with the PKC/PPARγ signalling pathway.^2^ PPAR‐γ acts as a transcriptional regulator of genes encoding proteins involved in lipid regulation,^36^ and its phosphorylation is mediated by PKC.^25^, ^35^, ^37^ In an in vitro study, the polyphenol quercitrin inhibited CD36 and SRA expression in ox‐LDL‐exposed macrophages by interfering with PKC/PPARγ signalling.^38^ Therefore, we also detected the levels of PPARγ in NPY‐induced foam cell formation. Our results showed that NPY could upregulate the expression of PPARγ via PKC activation, and this effect was inhibited by both Y1 and Y5 antagonists and a PKC inhibitor. The expression of CD36 and SRA was also decreased to some degree after treatment with Y1 and Y5 antagonists and PKC inhibitors.

**FIGURE 5:**
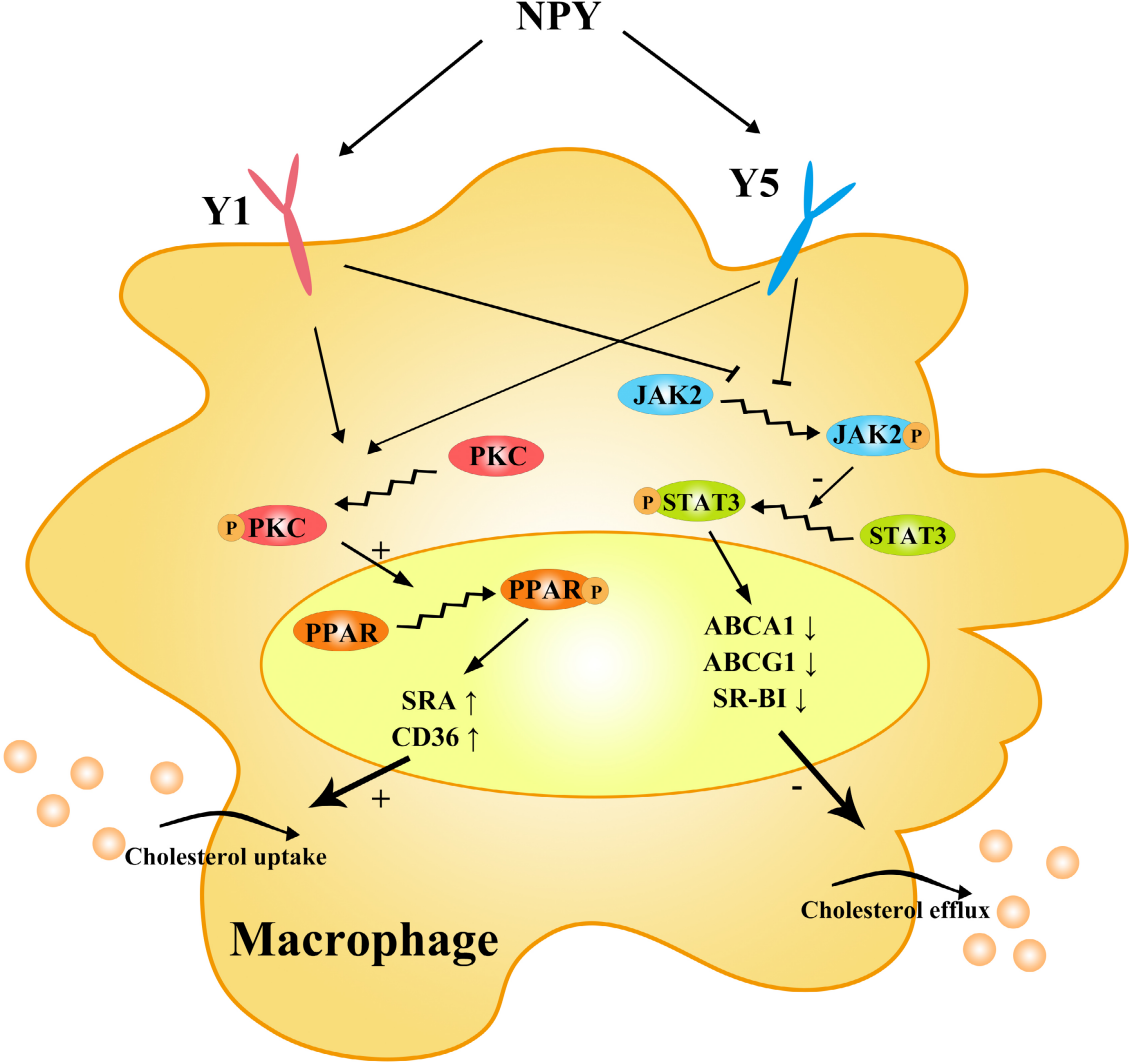
Schematic model of the effects of NPY on macrophages at cellular level

Inhibition of cholesterol efflux from macrophages is the main process that inhibits foam cell formation, which requires the activity of membrane transporters such as ABCA1, ABCG1 and SR‐BI.^12^, ^13^, ^14^, ^15^ First, we detected the efficiency of NPY on cholesterol efflux under ApoA1 and HDL. Our results indicate that NPY is an inhibitor of ApoA1‐ and HDL‐specific cholesterol efflux. Second, we investigated whether NPY could decrease cholesterol efflux from macrophages by inhibiting membrane transporter expression. We found an obvious reduction in ABCA1 and ABCG1 in the ox‐LDL + NPY treatment group compared to the ox‐LDL treatment group. As mentioned above, NPY‐activated Y1 and Y5 receptors play a key role in mediating cholesterol transport in ox‐LDL‐induced macrophages, and this effect can be similarly prevented by Y1 and Y5 antagonists. Studies have proven that JAK 2/STAT3 activation induces upregulation of ABCA1 and ABCG1 expression, which increases cholesterol export in macrophages.^26^, ^39^, ^40^ In this study, decreased expression of ABCA1, ABCG1 and SR‐BI by NPY was followed by downregulation of the JAK/STAT3 signalling pathway. This effect could be reversed to some degree by Y1 and Y5 antagonists. Taken together, as shown in Figure 5, the above data suggest that NPY binding to the Y1 and Y5 receptors inhibits the expression of ABCA1, ABCG1 and SR‐BI and cholesterol efflux through the JAK/STAT3 pathways.

Foam formation is the central link in atherosclerosis and previous studies have already demonstrated that sympathetic nerve activation is a high‐risk factor in plaque development. NPY as a neurotransmitter secreted by the sympathetic nerve has been proven to be a promoter in atherosclerotic plaque formation and the mechanisms may be very complicated. This study was mainly focused on the in vitro transport of ox‐LDL in macrophages which may exit some limitations given the complex process of cholesterol uptake and cholesterol efflux. However, our results revealed the potential mechanisms of NPY in regulating cholesterol transport that provided theoretical basis for further studies.

## AUTHOR CONTRIBUTIONS

**Yu Cai:** Conceptualization (equal); funding acquisition (equal); validation (equal); writing – original draft (equal); writing – review and editing (equal). **Zhengchao Wang:** Data curation (equal); formal analysis (equal); investigation (equal); methodology (equal); software (equal); writing – original draft (equal); writing – review and editing (equal). **Lun Li:** Investigation (equal); methodology (equal); resources (equal); writing – original draft (equal); writing – review and editing (equal). **Li He:** Conceptualization (equal); project administration (equal); resources (equal); software (equal). **Xinying Wu:** Data curation (equal); writing – review and editing (equal). **Mingjing Zhang:** Methodology (equal); software (equal). **Pengfei Zhu:** Data curation (equal); funding acquisition (equal); project administration (equal); supervision (equal); writing – review and editing (equal).

## FUNDING INFORMATION

This study was funded by the National Natural Science Foundation of Wuhan (Grant No. WX19Q24), the Health Commission of Hubei Province Scientific Research Project (No. WJ2019H371) and the National Natural Science Foundation of Hubei (No. 2019CFB806).

## CONFLICT OF INTEREST

The authors declare that they have no conflict of interest.

## Data Availability

The datasets used and/or analyzed during the current study are available from the corresponding author on reasonable request.
